# Gafas de realidad virtual como ayuda de distracción y disminución de la ansiedad en niña de 7 años que se somete a procedimiento de extracción dental. Reporte de caso

**DOI:** 10.21142/2523-2754-1101-2023-146

**Published:** 2023-03-26

**Authors:** Sandra Viviana Cáceres Matta, Valeria Isabel Trigos León, Luis Eduardo Carmona Arango

**Affiliations:** 1 Grupo de Investigación PROMOUC, Programa de Odontología, Facultad Ciencias de la Salud de la Universidad del Sinú Seccional Cartagena. Cartagena, Colombia. scaceres@unisinucartagena.edu.co Universidad del Sinú Grupo de Investigación PROMOUC, Programa de Odontología Facultad Ciencias de la Salud Universidad del Sinú Seccional Cartagena Cartagena Colombia scaceres@unisinucartagena.edu.co; 2 Programa de Odontología, Facultad Ciencias de la Salud de la Universidad del Sinú Seccional Cartagena. Cartagena, Colombia. Vaistrilen04@gmail.com Universidad del Sinú Programa de Odontología Facultad Ciencias de la Salud Universidad del Sinú Seccional Cartagena Vaistrilen04@gmail.com Cartagena Colombia; 3 Grupo de Investigación PROMOUC, Facultad de Odontología, Universidad de Cartagena. Cartagena, Colombia. lcarmonaa@unicartagena.edu.co Universidad de Cartagena Grupo de Investigación PROMOUC Facultad de Odontología Universidad de Cartagena Cartagena Colombia lcarmonaa@unicartagena.edu.co

**Keywords:** comportamiento, niños, ansiedad dental, gafas de realidad virtual, behaviour, kids, dental anxiety, virtual reality glasses

## Abstract

La realidad virtual tiene un enorme potencial para el tratamiento de la fobia a los tratamientos dentales en la odontopediatría. Esta tecnología también es prometedora porque muestra resultados satisfactorios y, en el área quirúrgica, tiene un alto potencial para tratamientos complejos, que permite resultados predecibles y seguros. Sin embargo, los estudios futuros deberían centrarse en establecer estándares tecnológicos con alta calidad de datos y en el desarrollo de aplicaciones aprobadas para la rutina clínica. La distracción es una técnica de manejo del dolor no farmacológica, comúnmente utilizada por los odontopediatras para controlar el dolor y la ansiedad. Hay algunas técnicas nuevas que utilizan la estimulación de audio y video, y distraen al paciente exponiéndolo a videos tridimensionales; estas técnicas se denominan sistemas audiovisuales de realidad virtual. El objetivo de este reporte de caso fue evaluar la efectividad de los anteojos de realidad virtual como una distracción para reducir la ansiedad en una niña de 7 años que acudió a la consulta de odontopediatría para un procedimiento de extracción dental.

## Abstract (en)

Virtual reality in Pediatric Dentistry has enormous potential, in the treatment of phobia of dental treatments, this technology is also promising, since it shows satisfactory results and in the surgical area it has a high potential for complex treatments, allowing predictable and safe results. However, future studies should have to establish technological standards with high data quality and in the development of applications approved for clinical routine. Distraction is a non-pharmacological pain management technique commonly used by Pediatric Dentists to control pain and anxiety. There are some new techniques that use audio and video stimulation and distract the patient by exposing him to three-dimensional videos. These techniques are called virtual reality audiovisual systems. The objective of this case was to evaluate the effectiveness of virtual reality glasses as a distraction aid to reduce anxiety in a 7-year-old girl who attends the pediatric dentistry office to perform a dental extraction procedure.

## INTRODUCCIÓN

La ansiedad dental se define como “un temor o temor anormal de visitar al odontopediatra para recibir una atención preventiva o terapia, esta ansiedad es injustificada en la mayoría de procedimientos dentales” y puede tener consecuencias psicológicas, cognitivas y conductuales^1^. Existe una proporción considerable de niños poco cooperativos durante las visitas al odontopediatra. La presencia de ansiedad en los niños sugiere que el sistema nervioso es vulnerable a estímulos nocivos durante el desarrollo; por ello, aliviar la ansiedad del niño ante el tratamiento dental es importante no solo para mitigar el miedo inmediato, sino también para evitar que la aprensión continúe en la edad adulta^2^. Se han propuesto estrategias de manejo para reducir la angustia durante el tratamiento dental en niños y se dividen principalmente en dos grandes categorías. La primera consta de técnicas conductuales que incluyen la técnica de decir-mostrar-hacer, distracción, inspiración, modelado e hipnotismo. La segunda consiste en técnicas farmacológicas^3^. La técnica de la distracción ha demostrado ser efectiva entre los niños para reducir la ansiedad usando gafas de realidad virtual^4^.

La distracción es una técnica útil para desviar la atención del paciente de lo que puede percibirse como un procedimiento desagradable, esto permite disminuir la percepción de lo desagradable y evitar el comportamiento negativo en el paciente durante la consulta de odontopediatría. Algunos investigadores sugirieron la distracción como un mecanismo de afrontamiento cognitivo que redirige pasivamente la atención del sujeto o involucra activamente al sujeto en la tarea^5^^-^^10^. Darle al paciente un breve descanso durante un procedimiento estresante puede ser un uso eficaz de la técnica de distracción antes de considerar técnicas de orientación conductual más avanzadas. Estudios en odontología recientes han demostrado que la distracción es una técnica común utilizada para reducir la reacción de dolor durante procedimientos invasivos cortos^6^^-^^10^.

De acuerdo con lo anterior, la distracción se clasifica en técnicas de distracción activa y pasiva. Las formas activas promueven la participación del niño en una actividad durante un procedimiento dental. Estas técnicas involucran varios componentes sensoriales del niño. Algunas de las formas empleadas son juguetes interactivos, cantar canciones, apretar pelotas, respiración controlada, imaginario guiado y relajación. Las formas pasivas, por otro lado, requieren que el niño permanezca tranquilo y silencioso durante un procedimiento en la consulta de odontopediatría. En este caso, la distracción se logra mediante la observación de la actividad o estímulo en lugar de su participación abierta, que incluye técnicas auditivas y audiovisuales. Hay varias opciones tecnológicas disponibles para la distracción tanto visual como auditiva, como música de fondo, televisores, juegos de computadora y anteojos de realidad virtual (VR) como los utilizados en este reporte de caso^11^^,^^12^.

En los últimos años, ha habido un aumento en la investigación del comportamiento en la realidad virtual y el mundo virtual. Al animar a un paciente a concentrarse su atención en otros pensamientos, hay menos atención disponible para el dolor y la ansiedad en la consulta odontológica. La realidad virtual utiliza tecnologías avanzadas para crear entornos virtuales que permiten a los pacientes sumergirse en un mundo interactivo y simulado. Estos sistemas avanzados interactúan en muchos niveles con el entorno virtual, estimulando imágenes, sonidos y movimientos para alentar la inmersión en el mundo virtual y mejorar la distracción del dolor. Se han realizado muy pocos estudios de realidad virtual en odontopediatría y reportes de casos para controlar la ansiedad. Algunos estudios concluyeron que el uso de la distracción audiovisual logra disminuir no solo la ansiedad, sino también la percepción del dolor^13^. Por lo tanto, el objetivo de este caso fue evaluar la efectividad de los anteojos de realidad virtual como una distracción para reducir la ansiedad en una niña de 7 años que acudió a la consulta de odontopediatría para un procedimiento de extracción dental.

## REPORTE DE CASO

Paciente de sexo femenino de 7 años, acudió a la consulta de odontopediatría en la facultad de Ciencias de la Salud de la Universidad del Sinú Seccional Cartagena, unidad académica que desempeña actividades educativas dentro de los lineamientos, políticas y criterios de formación de profesionales del área de la salud.

Se obtuvieron el consentimiento y el asentamiento informado, además de los permisos para el uso fotográfico y divulgación de información con fines académicos investigativos, cumpliendo la política de Ética, Bioética e Integridad Científica del Ministerio de Ciencia, Tecnología e Innovación (Minciencias).

La niña presentaba múltiples procesos cariosos; según clasificación de ICDAS, caries tipo 6. Fue considerada una paciente de difícil manejo en las primeras citas de odontopediatría, por su actitud negativa. Requirió un tratamiento que necesitaba la administración de un anestésico local, y la realización de procedimiento de extracciones dentales durante los meses de octubre y noviembre del 2022, por lo cual, inicialmente, se realizaron técnicas de adaptación decir-mostrar-hacer, sin obtener resultados positivos para los procesos de operatoria dental y adaptación para procedimientos de anestesia y extracciones dentales. Por ese motivo, se decidió implementar el uso de gafas de realidad virtual en la cuarta cita.

**Figura 1:**
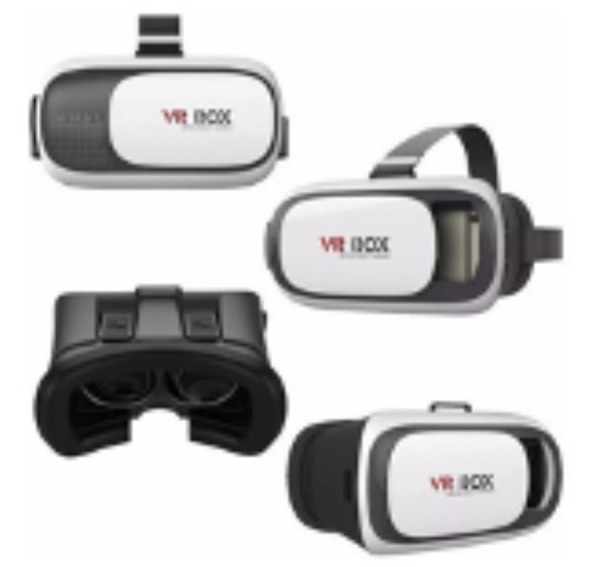
Auriculares BlackBugVirtual Reality Glasses 3DVR Box para teléfonos móviles de 4,7 a 6 pulgadas, modelo a236, India

El tratamiento consistió en cuatro sesiones consecutivas de adaptación. Todos los procedimientos dentales fueron realizados por una odontopediatra con experiencia y con la ayuda de una estudiante de ciclo final del Programa de Odontología de la Universidad del Sinú (Cartagena). En la primera consulta, se le colocó anteojos de realidad virtual, utilizando la técnica decir-mostrar-hacer, y se le realizó profilaxis oral. En la segunda visita se tomó aproximadamente 20 minutos para que la paciente se acostumbrará a las gafas de realidad virtual (auriculares BlackBug Virtual Reality Glasses 3D VR Box para teléfonos móviles de 4,7 a 6 pulgadas, número de modelo: a236, India) realizando procedimientos de operatoria dental y adaptación a ruidos, como la pieza de alta odontológica; esto con el fin de que durante los procedimientos de odontología se bloqueara por completo el campo visual de la niña. El dispositivo presentaba auriculares incorporados para emitir el sonido, a fin de cumplir con la técnica completa. El instrumento se conectó al dispositivo móvil (Apple iPhone) (Fig. 1), capaz de reproducir archivos audiovisuales MP4. Se reprodujo un solo episodio de la serie de dibujos animados durante toda la consulta. Una vez que se aseguró un dispositivo de realidad virtual en los ojos del niño, se comenzó a reproducir la caricatura. Finalizado el procedimiento, se retiraron los lentes.

**Figura 2:**
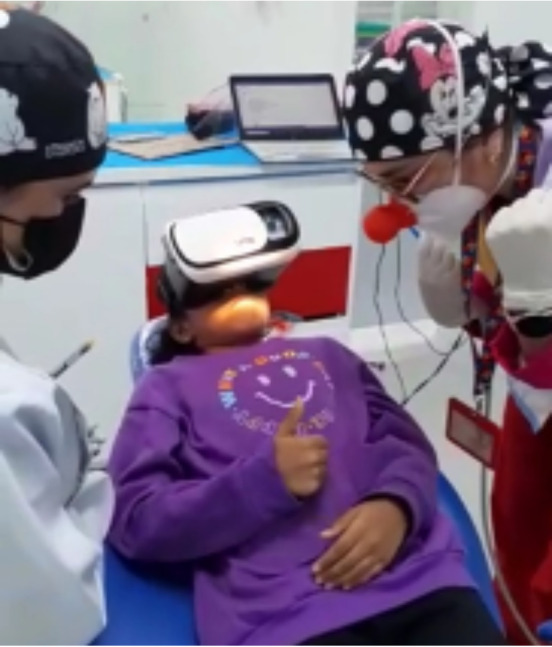
Paciente con gafas de realidad virtual, se logra adaptar a la paciente y disminuir la ansiedad en la consulta de odontopediatría.

**Figura 3:**
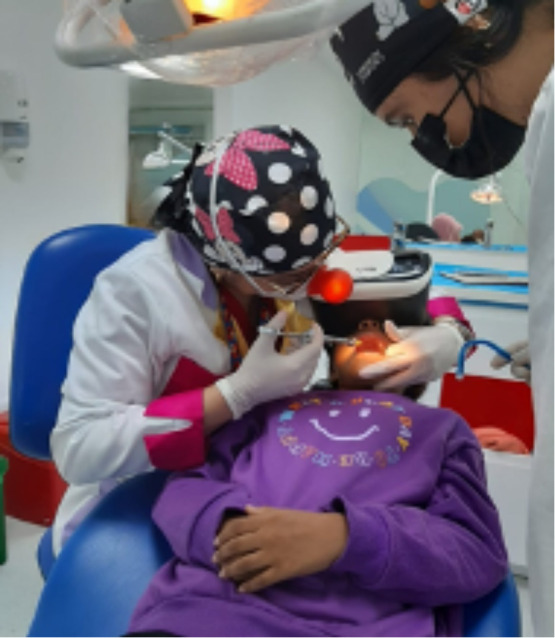
Odontopediatra aplicando la técnica anestésica para la realización del procedimiento de extracción dental. La paciente se encuentra totalmente adaptada y tranquila.

**Figura 4:**
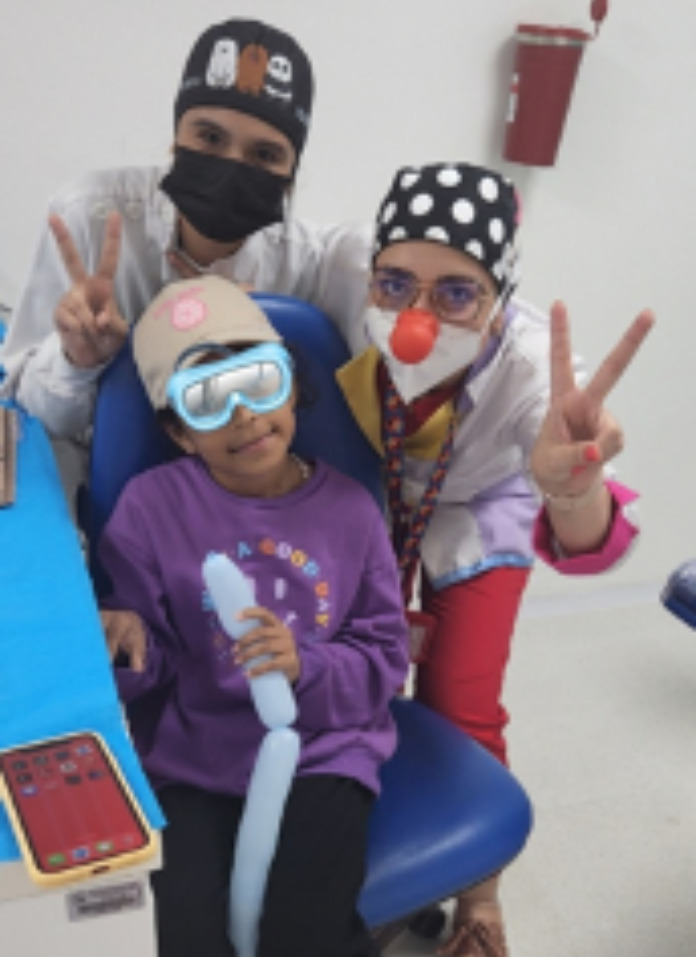
Paciente tras el procedimiento de extracción con actitud positiva y tranquila.

En la tercera consulta, se dio aproximadamente 10 minutos para que la paciente se acostumbrara a las gafas de realidad virtual (auriculares BlackBug Virtual Reality Glasses 3D VR Box para teléfonos móviles de 4,7 a 6 pulgadas, número de modelo: a236, India) realizando procedimientos de operatoria dental y adaptación a ruidos como la pieza de alta odontológica, esto con el fin de que durante los procedimientos de odontología se bloqueara por completo el campo visual de la niña. El dispositivo se conectó al dispositivo móvil reproduciendo los archivos audiovisuales MP4, se reprodujo un solo episodio de la serie de dibujos animados durante toda la consulta. Una vez que se aseguró un dispositivo de realidad virtual en los ojos de la paciente, se comenzó a reproducir la caricatura. Luego, se aplicó un agente anestésico tópico y se administró la anestesia local necesaria, para continuar con la extracción del órgano dentario 7.5, indicado para extracción por proceso de caries avanzado con compromiso periodontal no restaurable. La técnica empleada para la administración de anestesia local en el maxilar arco fue la de infiltración, y la técnica de bloqueo del nervio alveolar inferior se utilizó en el caso de la arcada inferior izquierda. Se procedió a verificar el éxito de la técnica anestésica y se procedió a realizar la extracción del órgano dentario instaurando el protocolo indicado para el caso, con total éxito del proceso (Figs. 2-4). 

**Figura 5:**
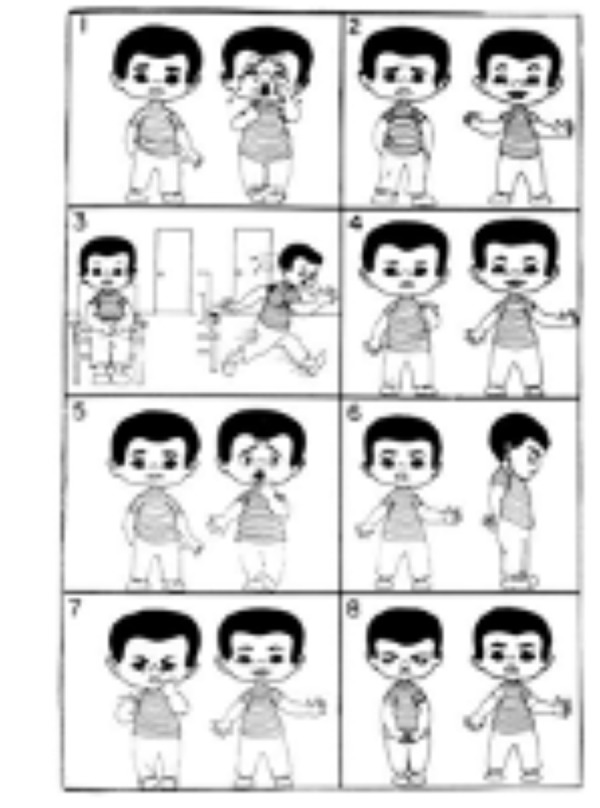
Prueba de imagen de Venham utilizada para la ansiedad.

En la cuarta visita, a los 7 días de realizado el procedimiento de control de cicatrización de la extracción dental, la paciente estuvo positiva y tranquila en la consulta odontopediátrica. El nivel de ansiedad de la niña en cada visita de procedimiento se evaluó mediante una combinación de tres medidas. La primera fue la prueba de imagen de Venham (VPT) (Fig. 5), que es una prueba proyectiva, psicométrica y de automedición que se utiliza para medir el estado de ansiedad del niño pequeño. Se compone de ocho cartas, con dos dibujos en cada una, una figura ansiosa y una no ansiosa. Se le pidió a la niña que seleccionará las figuras que más le parecían en ese momento. Todas las tarjetas fueron mostradas con número y ordenadas en consecuencia. Si la niña seleccionaba una figura ansiosa, entonces se registraba una puntuación de 1; si la niña elegía una figura no ansiosa, se registraba una puntuación de 0. La escala tenía un rango de 0 a 8. Asimismo, se midieron la frecuencia del pulso y la saturación de oxígeno usando el oxímetro de pulso de dedo, que es una medida directa de la excitación fisiológica, pues su aumento se atribuye al estrés durante los procedimientos dentales.

## DISCUSIÓN

Los procedimientos dentales, en particular la administración de anestesia local, son a menudo una fuente de ansiedad y estrés para los niños, lo que a su vez aumenta la reactividad al dolor. Por lo tanto, es muy importante utilizar intervenciones específicas para distraerlos de los procedimientos del tratamiento dental. La distracción, uno de los enfoques psicoconductuales utilizados en situaciones de tratamiento médico y dental, se define como un enfoque no aversivo utilizado para reducir la incomodidad de un niño al desviar su atención de la tarea principal, a fin de lograr un tratamiento exitoso con alta calidad^1^^,^^2^. Por lo tanto, este caso probó la efectividad de la distracción usando anteojos VR en los niveles de ansiedad de una niña de 7 años sometida al procedimiento de extracción dental. La ansiedad se midió por medios tanto objetivos como subjetivos. La medida subjetiva la hizo la foto de Venham. La medida objetiva de la ansiedad se evaluó midiendo la frecuencia del pulso y la saturación de oxígeno con la ayuda de un oxímetro de pulso. La frecuencia del pulso está gobernada por el sistema nervioso, especialmente el sistema nervioso autónomo (SNA) que refleja las emociones negativas en términos de parámetros fisiológicos, como la frecuencia cardíaca, la respiración y la temperatura corporal. Por lo tanto, las respuestas fisiológicas del SNA son indicadores que se utilizan para saber si una persona está estresada o relajada^4^^,^^5^.

Los estudios han llegado a la conclusión de que el uso de la distracción audiovisual durante el tratamiento dental fue más efectivo para controlar a los niños ansiosos que el uso exclusivo de distracción auditiva. Estos beneficios pueden estar relacionados con imágenes más inmersivas debido a los auriculares oclusivos que proyectan las imágenes justo en frente de los ojos del usuario y bloquean los estímulos del mundo real. La atención del niño se centra en lo que sucede en el mundo virtual más que en el entorno que lo rodea^7^. La aplicación de la distracción VR se basa en la suposición de que la percepción del dolor tiene un gran componente psicológico y que el dolor atrae una fuerte respuesta de atención que provoca ansiedad. Recientemente, también se descubrió que la realidad virtual cambia la forma en la que las personas interpretan las señales de dolor entrantes y, de hecho, reduce la cantidad de actividad cerebral relacionada con el dolor^9^^,^^10^.

Estas tecnologías no solo se aplican en el campo profesional, sino que han mejorado todos los campos de nuestra vida^3^. En el campo dental, la implantología y la cirugía ortognática son las áreas que tienen la mayor frecuencia de aplicación de RV mediante la planificación virtual^4^. Se evaluó el uso preoperatorio de los anteojos RV para mejorar la comprensión operativa en caso de un tercer molar profundamente impactado, una fractura de mandíbula inferior y una resección oncológica. Los autores señalaron que el examen preoperatorio con anteojos RV puede ayudar a comprender y planificar mejor el sitio quirúrgico, por ser una pieza innovadora de tecnología avanzada para mostrar datos anatómicos.

La realidad virtual es de creciente interés e importancia en la enseñanza de pregrado y posgrado en odontología, ya que ofrece conceptos de aprendizaje interactivo^12^. En la cirugía bucomaxilofacial, es una herramienta prometedora para procedimientos complejos que proporcionan resultados terapéuticos predecibles y seguros. Sin embargo, los ensayos clínicos con RV identificados en el campo de la odontología aún deben considerarse experimentales, ya que los dispositivos informáticos y el *software* de soporte son esenciales para su uso futuro en la práctica clínica^13^. Finalmente, en el mundo digitalizado de hoy, los niños se sienten atraídos por los juegos digitales, las series de dibujos animados, los teléfonos móviles, las tabletas digitales, etc. Usar anteojos VR como una distracción audiovisual es útil en el manejo de la ansiedad en niños temerosos durante los tratamientos dentales. Esta técnica de distracción también reduce el estrés operatorio en el dentista pediátrico. Por lo tanto, esta técnica se puede utilizar en la práctica pediátrica de rutina como complemento de otras técnicas de manejo del comportamiento.

## CONCLUSIONES

El presente caso confirma la eficacia de la distracción VR como medio de la técnica de manejo del comportamiento en nuestra práctica diaria. El uso de la RV como técnica de distracción mejora el parámetro fisiológico de los niños 7 años, pero no reduce la ansiedad autoinformada del paciente, según la prueba de imagen de Venham utilizada. La apariencia del equipo dental que provoca ansiedad y el hecho de que el niño se centre en todos los detalles del procedimiento es una de las razones más importantes del estrés asociado con los procedimientos dentales en los niños. Por lo tanto, los efectos positivos de la distracción VR sobre la ansiedad se atribuyen al bloqueo de los campos visuales de los niños y, como resultado, a una técnica de distracción exitosa. En nuestra era de odontología digital, el uso de una ayuda audiovisual puede ser un complemento para reducir la ansiedad infantil.

## Fuente de financiamiento:

Autofinanciado
